# Conspiratorial thinking as a precursor to opposition to COVID-19 vaccination in the US: a multi-year study from 2018 to 2021

**DOI:** 10.1038/s41598-022-22014-5

**Published:** 2022-11-03

**Authors:** Daniel Romer, Kathleen Hall Jamieson

**Affiliations:** Annenberg Public Policy Center, University of Pennsylvania, 202 S. 36th ST, Philadelphia, PA 19104 USA

**Keywords:** Psychology, Human behaviour, Medical research, Viral infection

## Abstract

Despite widespread availability of safe and effective COVID-19 vaccines in the US, only about 66% of the eligible US population had taken the recommended initial doses of the COVID-19 vaccines as of April 2022. Explanations for this hesitancy have focused on misinformation about the vaccines, lack of trust in health authorities, and acceptance of conspiracy theories about the pandemic. Here we test whether those with a conspiratorial mindset, which distrusts a wide range of institutions, were poised to reject COVID vaccines before the pandemic even began. To answer that question, we reinterviewed members of a national US panel that we had previously surveyed beginning in 2018. As hypothesized, having a conspiratorial mindset in 2019 predicted COVID-vaccination hesitancy in 2021 better than prior trust in health authorities or acceptance of vaccine misinformation. Those with the mindset were also more likely to consume media that bolstered belief in pandemic conspiracies. Research is needed on the determinants of conspiratorial mindset and ways to minimize the likelihood that consequential health decisions will be influenced by it.

## Introduction

Despite the widespread no-cost availability of COVID-19 vaccines in the US and evidence of their safety and efficacy from both clinical trials and the experience of the tens of millions of recipients^1^, only about 66% of the eligible US population had taken the recommended initial doses of the vaccine by the end of April 2022^2^. However, vaccination hesitancy, which the SAGE working group, a prominent board of medical experts, defined as “delay in acceptance or refusal of vaccination despite availability of vaccination services”^3^, was a matter of concern before the appearance of SARS-CoV-2^4^. In 2019, the World Health Organization characterized it as “one of the top global health threats”^5^.

Across the pre-pandemic decades, researchers had identified declining trust in government and health authorities as a factor in the erosion of support for vaccination^6–8^. Differences in trust across countries were a powerful predictor of engaging in preventive behavior during the first year of the COVID pandemic as well^9–12^. Closely associated with distrust is the acceptance of such misinformation about vaccination as the beliefs that the MMR vaccine causes autism in children^13–15^ and vaccines contain harmful ingredients such as antifreeze^16,17^. Persons holding such beliefs are also more likely to search for and use unapproved medical treatments^18–20^.

Another source of opposition to vaccination was conspiracy beliefs about the origin of the disease and vaccination against it^21–25^. Conspiracy theories reject established explanations for events and posit instead that malign actors are secretly responsible for them^26–28^. Experimental exposure to vaccination conspiracies has been shown to increase vaccination hesitancy^29^, a finding consistent with observational studies conducted during the pandemic^21,27,28^.

Because prior to the pandemic, the conspiratorial thinking style that underlies acceptance of conspiracy theories had been linked to anti-vaccination attitudes in both the US and other countries^17,30–32^, it is possible that rather than being affected by novel COVID-19-related conspiracy theories and associated misinformation about COVID vaccines, these newly emerging conspiracy theories confirmed pre-existing doubts about vaccination among persons with a conspiratorial mindset.

The conspiratorial mindset may not only have provided a backdrop for vaccination hesitance in the US, but may also have motivated increased exposure to conspiratorial messages about the pandemic^32,33^. Sources of such exposure included reports of claims by the 45th president of the US that the supposed deep state within his own government and malign forces in mainstream media were undercutting his ability to tame the pandemic^34^. Such attacks were among the factors politicizing the public’s response to it^35,36^. Individuals with a conspiratorial mindset were more likely to rely on social and conservative media that trafficked in pandemic conspiracy theories, including that the virus was created by the Chinese government as a bioweapon^22,32^. In addition, persons with a conspiratorial mindset in the US avoided more mainstream legacy media, such as broadcast television and national newspapers, the use of which was associated with less embrace of pandemic-related conspiracy theories^22^ and greater acceptance of COVID-19 vaccination^32^. These patterns of media use suggest that not only were persons with a conspiratorial mindset drawn to media that supported such thinking, but these same people displayed a lack of trust in mainstream media sources that tended to presuppose the value of vaccination.

Here we test a model of vaccine hesitancy that locates resistance to COVID-19 vaccination among those prone to conspiratorial thinking regarding the workings of the government and health authorities even before the pandemic arrived. Because conspiratorial thinking attributes malign motives to those in power^28,37,38^, the model proposes that by undermining trust in government institutions and authorities charged with protecting public health^23^, such thinking subverts acceptance of those authorities’ recommendations for action, such as receiving a vaccine^21^. At the same time, those with a conspiratorial mindset distrust mainstream media and are drawn to sources that reinforce rather than challenge conspiracy theories^14,39^, including media that promote misinformation about vaccines^14,22^ and unapproved forms of treatment and other health interventions^18–20^. We expected all these tendencies of a conspiratorial mindset to contribute to the hesitancy that has been observed for COVID-19 vaccines in the US.

Drawing on a large national probability sample empaneled in 2018 and reinterviewed early in 2021 shortly after vaccines were authorized for use in the US, we assessed whether conspiratorial thinking that was present a year before the outbreak of the COVID pandemic served as a major driver of opposition to COVID-19 vaccination, and if so, whether it was heightened by exposure to media that promoted conspiracies about the pandemic. This process allowed us to test our model’s major predictions that a prior conspiratorial mindset would better predict a range of problematic beliefs and attitudes in 2021 (i.e., lack of trust in health authorities, acceptance of conspiracy theories and misinformation about the pandemic and COVID vaccines, hesitance about COVID-19 vaccines, hesitance toward other vaccines, such as for the flu, and less concern about the health effects of COVID) than other predictors. It also allowed us to test the hypothesis that having this mindset predicts the use of conservative media that have been observed to promote conspiracies about the pandemic that undermine confidence in the government’s response to the crisis^16,32^.

To test the robustness of a conspiracy mindset, we assessed different ways of measuring the tendency. In the 2019 survey, belief in various famous conspiracy theories was assessed, such as one alleging that fluoridation of public water supplies was a plot to dispose of hazardous chemicals^17,40^. Conspiracy theories such as these reflect lack of trust in the US government, especially pertaining to health. In 2021, we used a measure that assesses more generic beliefs about how important events are controlled by secretive and malevolent forces^41,42^ which predicted belief in COVID conspiracies in previous research^16,32^. Consistent with previous research^41,43^, we expected that these alternative measures would be related and would demonstrate that this thinking style is more than just a summary of endorsements of various conspiracy theories^44^, especially as they relate to government policies.

Despite the likelihood that conspiratorial thinking is a stable individual characteristic, we also wanted to determine whether its relation to other individual differences might change over time. In particular, given the political polarization of vaccination and COVID-19 prevention measures such as masking and divergent patterns of media coverage about them, it is possible that supporters of President Donald Trump and those who were more likely to consume conservative media also became more attracted to conspiratorial thinking, while those on the other side of the partisan divide became less so. Such a finding would suggest that a conspiratorial mindset was subject to realignment consistent with the finding that individuals are more receptive to conspiracy theories about those holding political ideologies dissimilar from their own^45^.

We also recognize that the urgency of the pandemic and the heightened national attention given to vaccination may have changed how previous vaccine doubters felt about vaccination in general. We therefore also were interested in determining whether those demographic subgroups expressing vaccine hesitancy in 2019, such as those of older age, female gender, higher education, and Black and Hispanic identity, may have become more accepting of vaccination during the pandemic.

## Results

Table 1 shows the demographic distribution of the sample and the percentages of respondents who reported intentions to receive the COVID-19 vaccine in relation to those characteristics. All the demographic differences were related to vaccination intention by *X*^2^ tests, *p* < 0.001. The survey items that were hypothesized to measure various latent factors related to vaccine hesitancy are shown in Tables 2, 3, 4, 5 along with their loadings on the measurement model determined with confirmatory factor analysis. Further descriptions of the measures are detailed in the Materials and Methods. The measurement model fit the data well, with a RMSEA = 0.041 (90% CI = 0.040, 0.043), CFI = 0.91, and SRMR = 0.048. Although we expected that COVID vaccine conspiracy theories would be correlated with belief in vaccine misinformation, we found that acceptance of those theories loaded directly on an overall misinformation factor. In addition, beliefs about the harms of COVID vaccines also loaded directly on the misinformation factor, indicating that newly emerging concerns about vaccination were simply assimilated into pre-existing forms of misinformation about vaccines and vaccination in general.

**Table 1:** Demographic composition of the sample in 2021 and reported likelihood of COVID-19 vaccination with *X*^2^ tests of relationshjp.

Characteristic	Percent (N = 1243)	Likelihood of COVID-19 Vaccination				*X*^2^	*P* value
		Not likely (%)	Not too likely (%)	Somewhat likely (%)	Very Likely (%)		
**Age**						61.1^a^	< .001
18–24	1.6	20.0	15.0	20.0	45.0		
25–34	13.6	14.2	17.8	16.6	51.5		
35–54	34.4	18.9	19.4	23.0	46.5		
55–64	21.2	10.6	13.7	19.0	56.7		
65 +	29.1	4.7	8.6	15.3	71.4		
**Gender**						13.3^a^	< .001
Male	49.4	9.8	11.3	19.6	59.3		
Female	50.6	14.0	17.2	17.2	51.5		
**Education**						70.2^a^	< .001
HS grad or less	16.8	17.8	19.7	23.6	38.9		
Some college	31.2	16.0	17.6	19.9	46.5		
Bachelor’s degree	28.1	9.2	13.2	16.4	61.2		
Post-bachelor	23.9	5.7	7.4	15.2	71.6		
**Income**						40.1^a^	< .001
< $10 K	2.8	25.7	28.6	14.3	31.4		
$10 K to $40 K	25.3	15.7	18.2	20.8	45.4		
$40 K to $75 K	28.2	12.0	16.3	18.9	52.7		
$75 K to $100 K	15.8	10.2	10.2	16.3	63.3		
$100 K to $150 K	16.8	5.8	9.6	20.2	64.4		
$150 K or more	11.1	11.6	9.4	13.0	65.9		
**Race/Ethnicity**						54.5^b^	< .001
Non-Hispanic White	70.9	11.6	11.5	15.6	61.3		
Non-Hispanic Black	9.4	13.8	20.7	25.9	39.7		
Hispanic	13.0	13.7	24.2	24.8	37.3		
Other	6.7	9.6	15.7	25.3	49.4		
**Religion**						60.7^b^	< .001
Evangelical	25.4	18.7	21.3	21.9	38.1		
Other Christian	43.8	10.3	12.7	18.6	58.4		
Non-Christian	30.8	8.7	10.8	15.2	65.4		
**Political Ideology**						114.4^a^	< .001
Very Liberal	10.9	3.0	3.7	10.4	82.8		
Somewhat Liberal	20.9	2.7	12.1	15.2	70.0		
Moderate	39.9	13.0	13.6	22.6	50.7		
Somewhat Conservative	21.0	15.8	21.6	17.8	44.8		
Very Conservative	7.3	32.2	16.7	20.0	31.1		
Total	100	11.9	14.3	18.4	55.4		

*X*^2^ tests denoted with ^a^ refer to linear relations, while those with ^b^ are tests for categorical variables.

**Table 2:** Measures used to assess theoretically relevant predictors of vaccination intention in 2021 and standardized loadings on latent factors in measurement model.

Item	Loading
**Trust in Health Authorities (2021**): In general, how confident, if at all, are you that the following are providing the public with trustworthy information about the safety and effectiveness of the COVID-19 vaccines that are being distributed in the US? (Scale from 1 to 4: Not at all confident to Very confident)	
The Food and Drug Administration (FDA)	.870
The U.S. Centers for Disease Control and Prevention (CDC)	.804
Dr. Jerome Adams, the U.S. Surgeon General	.646
The pharmaceutical companies that produce vaccines	.778
**Perceived Risk of Covid Infection (2021):** How much of a risk to your health and well-being do you think the following activities are right now? (Scale from 1 to 4: Large risk to no risk)	
Attending in-person gatherings of friends and family outside your household	.832
Dining at a restaurant	.850
Spending more time inside public places as the weather turns colder	.783
Traveling for the holidays	.829
How worried, if at all, do you feel about the possibility that you or someone in your family will become infected with the coronavirus? (Scale from 1 to 4: Very worried to Not at all worried)	.587
**Vaccine Fears and Misinformation (2021):** Please indicate if you believe the statement is true, false, or if you aren’t sure. (Scale from 1 to 4: Definitely false to Definitely true; Not sure = missing)	
Vaccines in general are full of toxins and harmful ingredients like “antifreeze”	− .819
Vaccines given to children for diseases like measles, mumps, and rubella do not cause autism	.477
Vaccines approved for use in the U.S. are safe	.716
It makes no difference if parents delay the timing of vaccines instead of relying on the official CDC vaccine schedule	− .568
^a^The COVID-19 vaccine will change your DNA	− .723
^a^The pharmaceutical industry created the coronavirus to increase sales of its drugs and vaccines	− .712
^a^The vaccine against COVID-19 being developed with support by Microsoft founder Bill Gates contains microchips that can track the person who has been vaccinated	− .757
The Food and Drug Administration also known as the FDA has concluded that the Pfizer COVID-19 vaccine is safe and effective for adults	.593
Just your best guess, how effective is the typical flu vaccine in preventing a person from getting infected with the seasonal flu? (Scale from 1 to 3: Very effective to Not at all effective)	− .482
How likely, if at all, do you think it is that someone can get a serious case of the flu from the flu vaccine? (Scale from 1 to 3: Not at all likely to Very likely)	− .668
How likely, if at all, do you think it is that someone can get a serious case of COVID-19from a COVID-19 vaccine? (Scale from 1 to 3: Not at all likely to Very likely)	− .725
**Conspiracy Beliefs about COVID Pandemic (2021):** Please indicate if you believe the statement is true, false or if you aren’t sure. (Scale from 1 to 4: Definitely false to Definitely true; Not sure = missing)	
Some health officials at the U.S. Centers for Disease Control and Prevention, also known as the CDC, have exaggerated the danger posed by the coronavirus in order to damage the Trump presidency	.851
The coronavirus was created by the Chinese government as a biological weapon	.749
The U.S. Centers for Disease Control and Prevention, also known as the CDC, has admitted that most of the deaths attributed to COVID-19 were actually caused by other serious illnesses and not by the coronavirus	.826
The number of reported COVID-19 deaths is higher in the U.S. than in other countries because U.S. doctors and hospitals receive extra compensation when a doctor reports that a patient died of COVID-19	.793

^a^These items assessed conspiracies regarding the Covid vaccine but loaded directly on the vaccine misinformation factor.

**Table 3:** Measures of trust, misinformation, and perceived risk of measles in 2019.

Item	Loading
**Trust in Health Authorities (2019**): How much trust, if any, do you have in the following institutions when it comes to addressing issues of public health? (Scale from 1 to 4: A great deal of trust to Very little trust at all)	
The U.S. Centers for Disease Control and Prevention, also known as CDC	.860
Your state government	.543
Your physician	.577
Pharmaceutical companies	.418
How much trust, if at all, do you have in the following to give you accurate information about the benefits and risks of vaccination? (Scale from 1 to 4: A great deal of trust to Very little trust at all):	
The U.S. Centers for Disease Control and Prevention	.901
Your primary care doctor or primary medical provider	.684
**Vaccine Fears and Misinformation (2019):** For each statement below, please indicate how accurate you think it is. (Scale from 1 to 4: Definitely false to Definitely true; Not sure = missing)	
Vaccines given to children for diseases like measles, mumps, and rubella can cause neurological disorders like autism	.701
Vaccines in general are full of toxins and harmful ingredients like ‘antifreeze’	.726
Just your best guess, how risky, if at all, do you think the measles, mumps, and rubella (MMR) vaccine is? (Scale from 1 to 4: Very risky to Not risky at all)	− .559
Just your best guess, please indicate how effective, if at all, you think the measles, mumps, and rubella (MMR) vaccine will be at preventing measles among those who get the vaccine in the future? (Scale from 1 to 4: Very effective to Not effective at all)	707
Based on what you know, is the measles, mumps, and rubella (MMR) vaccine? (Scale from 1 to 5: Much more risky than catching measles to Much less risk than catching measles)	.669
Based on what you know, how positive or negative do you feel about the measles, mumps, and rubella (MMR) vaccine? (Scale from 1 to 4: Very positive to Very negative)	.721
**Perceived Risk of Measles (2019)**	
How afraid, if at all, do you feel about measles? (Scale from 1 to 4: Very afraid to Not afraid at all)	.769
How disgusted, if at all, do you feel about measles? (Scale from 1 to 4: Very disgusted to Not disgusted at all)	.563
How concerned, if at all, are you that measles will become widespread throughout the United States? (Scale from 1 to 4: Not at all concerned to Very concerned)	− .597

**Table 4:** Measures of conspiratorial thinking at both times.

Item	Loading
**Conspiratorial Thinking Scale (2021):** Please indicate how much you agree or disagree with each of the following statements. (Scale from 1 to 5: Strongly agree to Strongly disagree)	
Much of our lives is controlled by plots hatched in secret places	.841
Even though we live in a democracy, a few people will always run things anyway	.416
The people who really ‘run’ the country are not known to the voters	.621
**Conspiratorial Beliefs Scale (2019):** Please indicate how much you agree or disagree with each of the following statements. (Scale from 1 to 5: Strongly agree to Strongly disagree)	
The Food and Drug Administration is deliberately preventing the public from getting natural cures for cancer and other diseases because of pressure from drug companies	.746
Public water fluoridation is really just a secret way for chemical companies to dump the dangerous byproducts of phosphate mines into the environment	.790
Certain U.S. government officials planned the attacks of September 11, 2001, because they wanted the United States to go to war in the Middle East	.727

**Table 5:** Measures of media use at both times.

Item	Loading
**Media Use (2021) (Only single items were used)** How much information do you get from sources such as? (Scale from 0 to 5: No information to A lot of information):	
ABC News, CBS News, or NBC News (Mainstream TV)	
Facebook, Twitter or YouTube (Social Media)	
The Associated Press, The New York Times, or The Washington Post (Mainstream Print)	
Local TV, radio, print, or local social media sites	
Fox News Channel or Rush Limbaugh (Conservative Media)	
Newsmax, One America News (OAN), Gateway Pundit, or Parler (Ultra-Conservative Media)	
**Alternative Health Media Use (2019) Principal Component Score** How often do you get information from each of the following sources? (Scale from 1 to 4: Regularly to Never):	
Online health blogs like Food Babe	.693
Social media accounts dedicated to alternative health, such as Natural News or Earth Clinic	.733
Television shows such as Dr. Oz and The Doctors	.708

We also identified some additional correlations between items that likely reflected similarities in wording that crossed either within or between factors. For example, the item concerning autism as a consequence of the MMR vaccine was correlated across the two survey years beyond what the underlying factors indicated. These correlations were added to the measurement model and were retained in all subsequent analyses to provide an adequate accounting of the relations between the various indicators.

**Figure 1:**
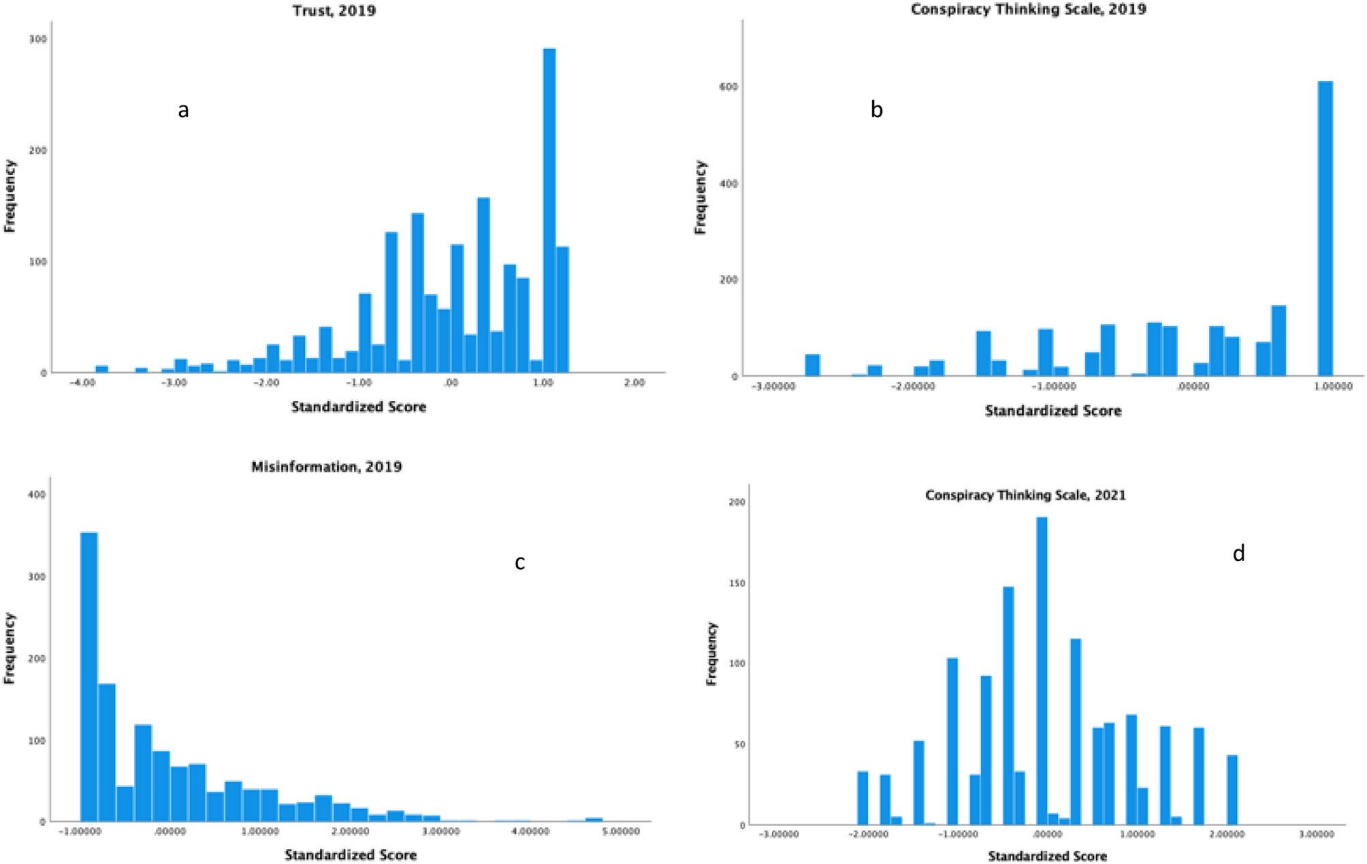
(**a**) Trust of health authorities, 2019; (**b**) conspiracy thinking, 2019; (**c**) misinformation, 2019; (**d**) conspiracy thinking, 2021.

The distributions of the three major predictors, trust, conspiracy thinking, and vaccine misinformation in 2019, as indexed by the first principal component of each set of scores are in Fig. 1a–c. All three scores were skewed such that distrust, belief in conspiracy theories, and misinformation were less frequent than the opposite tendencies. If we use scores greater than one standard deviation from the mean as a cutoff, then about 20% of the sample had high conspiracy thinking scores, about 14% of the sample had low trust in 2019 and about 16% had high misinformation.

The correlations between the factors in the measurement model as well as with COVID-19 vaccination intentions in 2021 are shown in Table 6. All factors associated with vaccination intention in 2021 were also related to similar measures in 2019, including conspiratorial thinking which was highly correlated across time (0.537). Conspiratorial thinking in 2019 was also correlated at 0.619 with belief in misinformation, − 0.621 with trust, and − 0.369 with intention to receive the flu vaccine in 2019. These relations show that conspiratorial thinking in 2019 was highly related to factors that have predicted hesitancy toward COVID-19 vaccines in 2021^16^. As we show below, there is evidence that conspiratorial thinking also became more aligned with other correlates in 2021 than it had been in 2019. It is also noteworthy that conspiratorial thinking in 2019 was more highly related to three out of four predictors of vaccination in 2021 than it was to those variables in 2019. This is as would be expected if it predicts those outcomes over and above its contemporaneous relations.

**Table 6:** Table of intercorrelations between current and lagged predictors of COVID vaccination intention based on the measurement model.

Factor	1	2	3	4	5	6	7	8	9	10
(1) COVID vaccination										
(2) Misinformation 2021	− .730									
(3) Trust 2021	.638	− .645								
(4) Risk Perception 2021	.461	− .413	.346							
(5) Conspiracy Mindset 2021	− .556	.704	− .563	− .346						
(6) Flu Intention 2021	.591	− .521	.420	.349	− .407					
(7) Misinformation 2019	− .502	.740	− .449	− .196	.473	− .458				
(8) Trust 2019	.492	− .649	.633	.283	− .519	.462	− .753			
(9) Risk Perception 2019	.179	.072	.141	.289	.004	.169	.183	.195		
(10) Conspiracy Mindset 2019	− .453	.669	− .426	− .135	.537	− .399	.619	− .621	− .014	
(11) Flu Intention 2019	.487	− .437	.368	.278	− .343	.691	− .484	.526	.276	− .369

**Figure 2:**
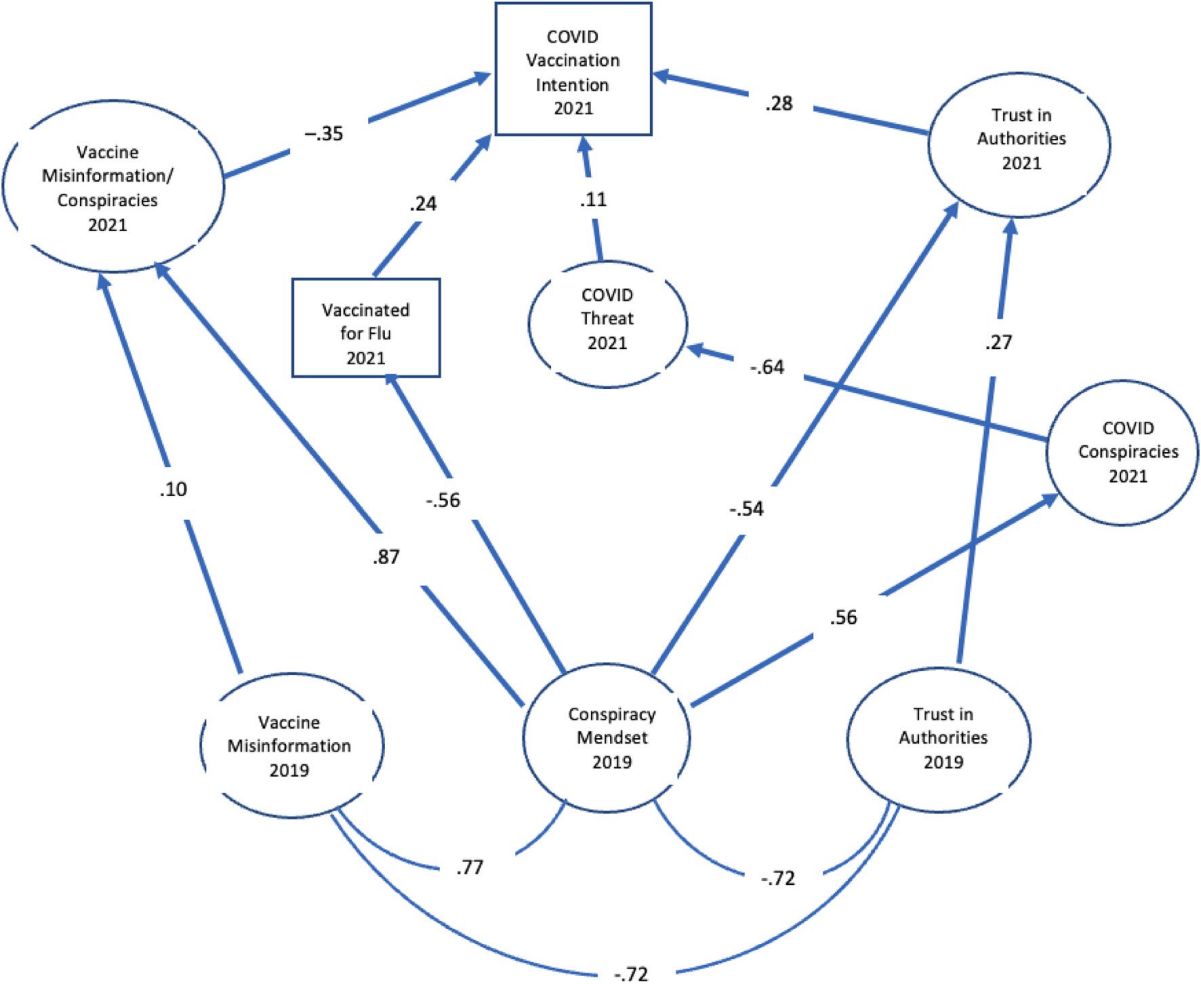
Structural relations in main model.

**Figure 3:**
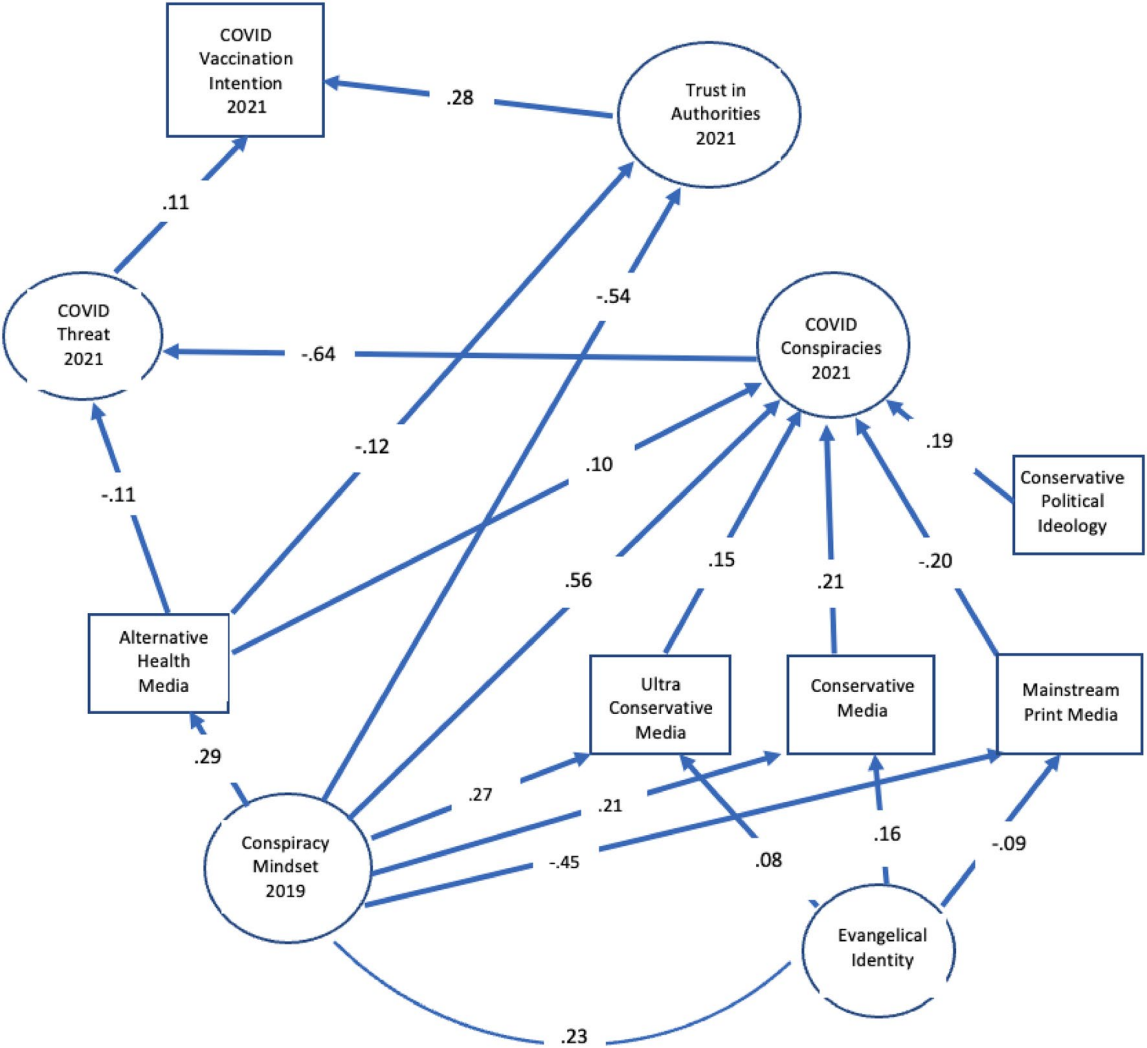
Structural relations for media predictors.

The final structural model trimmed for relations that were not within 90% confidence intervals (CIs) for parameters is shown in Figs. 2 and 3. Table 7 has the standardized path weights and CIs for all direct predictors in the model. To simplify the presentation of the model, we show the relations between conspiracy mindset in 2019 as it predicted the various indicators of COVID-vaccine hesitancy in Fig. 2. Relations between the mindset and various media uses are in Fig. 3.

**Table 7:** Standardized path weights and 99% confidence intervals (CIs) for structural equation model of vaccination intention.

Dependent Variable/Predictor	Path weight	99% CI
**Vaccination Intention**		
Trust 2021	.280	.191, .364
Covid threat 2021	.110	.050, .177
Misinformation 2021	− .352	− .451, − .259
Flu vaccination 2021	.243	.168, .314
Age 2021	.028	− .023, .074
Evangelical Identity 2018	− .058	− .114, − .011
**Trust 2021**		
Trust 2019	.265	.141, .388
Conspiracy Mindset 2019	− .538	− .668, − .406
Alternative Health Media 2019	− .116	− .056, − .179
Education 2021	.063	.003, .130
**Covid threat 2021**		
Covid Conspiracy Beliefs 2021	− .643	− .716, − .573
Alternative Health Media 2019	− .110	− .173, − .049
Black Identity 2021	.141	.073, .210
Female Gender 2021	.145	.087, .209
**Misinformation 2021**		
Misinformation 2019	.100	.024, .198
Conspiracy Mindset 2019	.873	.768, .994
**Flu Vaccination**		
Conspiracy Mindset 2019	− .557	− .621, − .493
**Covid Conspiracy Beliefs 2021**		
Conspiracy Mindset 2019	.556	.489, .626
Conservative Media 2021	.213	.148, .281
Ultra-Conservative Media 2021	.150	.083, .218
Mainstream Print 2021	− .201	− .254, − .143
Alternative Health Media 2019	.096	.053, .149
Conservative Political Identity 2021	.188	.124, .240
**Conservative Media 2021**		
Conspiracy Mindset 2019	.209	.141, .297
Evangelical identity 2018	.162	.084, .235
**Ultra-Conservative Media 2021**		
Conspiracy Mindset 2019	.271	.193, .369
Evangelical Identity 2018	.078	− .003, .163
**Mainstream Print 2021**		
Conspiracy Mindset 2019	− .445	− .509, − .379
Evangelical Identity 2018	− .087	− .149, − .017
**Alternative Health Media 2019**		
Conspiracy Mindset 2019	.294	.217, .371

The model provided an excellent fit to data as indicated by RMSEA = 0.042 (90% CI, 0.041, 0.044), CFI = 0.918, and SRMR = 0.048. It also accounted for 65% of the variation in vaccination intention. Support was found for the major hypothesis that in 2021, misinformation about vaccination, trust in health authorities, acceptance of the flu vaccine, and belief in pandemic-related conspiracies would be better predicted by 2019 conspiracy mindset than trust as measured in 2019, acceptance of vaccination misinformation in 2019, and demographic and political ideology differences. In addition, trust and misinformation as measured in 2019 did not add to prediction of these mediators other than to their respective measures in 2021. Conspiratorial thinking in 2019 also predicted reduced perceived personal threat of COVID in 2021 indirectly through its prediction of COVID-specific conspiracy beliefs (0.56 X − 0.64 = − 0.36). These relations supported the major hypothesis that conspiratorial thinking prior to the COVID pandemic prefigured major sources of resistance to vaccination for COVID in 2021.

One of the striking features of the structural model is the strong relation between conspiracy mindset in 2019 and belief in vaccine misinformation in 2021 (0.87). This relation indicates that although misinformation in 2021 included novel beliefs about the COVID vaccines, the tendency to accept such assertions was more strongly related to prior conspiracy mindset than to earlier acceptance of misinformation. The relation between misinformation and conspiracy mindset was also strong at both times (0.704 in 2021 and 0.619 in 2019 in Table 6). Other research has found that vaccine misinformation is a powerful predictor of COVID vaccination hesitancy^15,16^, but these results suggest that this is more a result of its underlying relation to conspiratorial thinking than to the specific misinformation that circulates at the time.

The final model also required inclusion of measurement error in responses to the conspiracy thinking items. The fact that these positive correlations between the items were orthogonal to the underlying factor suggests the presence of a response bias that reflects the social undesirability of expressing belief in the conspiracies, as has been previously observed^46^. Nevertheless, after removing those measurement biases, the underlying factor was strongly related to vaccination and media outcomes.

### Relations with media use

As predicted, conspiratorial thinking in 2019 also predicted types of media use in 2021 (Fig. 3) controlling for correlations with various demographic differences. Even though conspiratorial thinking was correlated with conservative political ideology (0.37), Evangelical (0.23) and Black (0.13) identity as well as other demographic differences [education (− 0.34), income (− 0.26), age (− 0.21) female gender (0.14) and Hispanic identity (0.09)], it nevertheless was independently and positively related to use of both kinds of conservative media in 2021 and inversely related to use of mainstream print media. These uses of media were also robust to inclusion of alternative health media, which also predicted belief in COVID conspiracies and less concern about the COVID health threat.

Although the mindset was related to use of social media and mainstream TV news, those uses of media were not directly related to COVID conspiracy beliefs apart from political ideology and use of the other media and thus are not shown in Fig. 3. Nevertheless, conspiratorial thinking in 2019 was indirectly related to belief in COVID conspiracies as mediated by the four uses of media (0.204). Interestingly, as we note below, use of conservative media also became more aligned with conspiratorial thinking over time (Table 8).

**Table 8:** Comparisons between correlations with predictors of COVID vaccination in 2019 and 2021 and demographic, political identity, and media use characteristics in 2018/19. Differences greater than 3 standard errors are highlighted.

Correlate	Vaccine Misinfor-mation 2021	Vaccine Misinfor-mation 2019	Trust in Health Authorities 2021	Trust in Health Authorities 2019	Risk of Covid 2021	Risk of Measles 2019	Conspiracy Mindset 2021	Conspiracy Mindset 2019	Flu Vaccination 2021	Flu Vaccination 2019
Age	**− .234**	**.157**	.178	.187	.171	− .045	− .165	− .204	.273	.197
Female	**− .125**	**− .017**	− .072	− .058	.095	.162	.034	.097	− .054	− .037
Education	**− .266**	**.201**	.114	.185	− .001	− .051	− .237	− .282	.158	.117
Income	**− .251**	**.177**	.135	.160	.006	− .077	− .234	− .304	.166	.118
Evangelical	.147	.141	− .077	− .061	− .058	− .050	.108	.046	.000	.025
Black Identity	**.139**	**− .124**	.004	− .007	.121	.064	.126	.123	− .015	− .029
Hispanic Identity	**.110**	**− .083**	− .028	− .016	.062	.048	.061	.124	− .042	.018
Conservative Ideology	**.336**	**− .233**	− .243	− .228	**− .429**	**.013**	**.314**	**.106**	− .149	− .133
Democrat	**− .261**	**.196**	.238	.221	**.358**	**.158**	**− .299**	**− .109**	.203	.163
Republican	**.213**	**− .100**	− .124	− .067	**− .301**	**− .006**	**.240**	**.005**	− .028	− .022
Print News	**− .313**	**.213**	.226	.238	**.252**	**.094**	− .277	− .229	.237	.202
TV News	**− .237**	**.142**	.268	.230	.217	.151	− .184	− .134	.241	.172
Conservative Media	**.176**	**− .100**	− .129	− .121	**− .315**	**− .021**	**.227**	**.033**	− .096	− .061
Ultra-Conservative Media (2021)	**.226**	**− .124**	− .224	− .172	**− .359**	**− .016**	**.253**	**.113**	− .135	− .064
Social Media	**− .129**	**.110**	.118	.053	.115	.065	− .081	− .026	.070	.056
Alt Health Media	.225	.252	**− .095**	**− .205**	.057	.106	.194	.269	− .143	− .068

The model included controls for demographic and political differences (see Table 7 for significant predictors of factors in the model). Although all those differences were related to COVID-19 vaccination intentions in 2021 (Table 1), the only demographic characteristic that was directly related to vaccination intention was Evangelical religious identity (− 0.06 in Table 7). Because that characteristic was also related to media use and conspiratorial thinking, we included it as a direct predictor of media use to see if that would account for its ability to directly predict vaccination. As seen in Fig. 3, Evangelical identity did predict media use in 2021 apart from conservative ideology and conspiratorial thinking. Nevertheless, it remained a small direct negative predictor of vaccination, suggesting that its influence was mediated by factors not in the model. Thus, the mediators of vaccination intention explained all the demographic and political ideology differences in the model except for religious identity.

### Alternative model for vaccination intention

We tested an alternative model in which the trust and misinformation factors in 2019 were allowed to predict the various mediators of vaccination intention, including media use, with conspiratorial mindset restricted to only correlate with misinformation and trust in 2019. This model produced a weaker fit by all criteria: RMSEA = 0.046 (90% CI, 0.044, 0.047), CFI = 0.903, SRMR = 0.058. In addition, the sample-size adjusted Bayesian fit criterion was greater for the alternative model: 129,280 versus 128,812. Examination of the residuals for the model indicated that it was less successful in explaining the relation between COVID conspiracy beliefs and misinformation than the model in Fig. 2 which fully accounted for the relation as a function of conspiratorial mindset. Thus, conspiratorial mindset explained why both types of beliefs were related, something that was less well captured by prior trust and misinformation alone.

### Changes in Associations over Time

We also were interested to see whether demographic and political ideology differences changed in relation to conspiratorial thinking and other predictors of vaccination across time. Because we had a different measure of conspiratorial thinking at each time point, we cannot assess absolute changes in this tendency. However, we can assess differences in the proportion of respondents who departed at least one standard deviation from the mean at both time points. As noted above, in 2019 about 20% of the panel was classified as high by this criterion. As seen in Fig. 1d, this proportion did not change appreciably, with about 19% meeting this criterion. This suggests that despite the differences in the distributions, there was not a major shift in the prevalence of conspiratorial thinking over the two years. However, there could have been shifts in how this tendency aligned with partisan differences.

The results in Table 8 support the occurrence of differential change based on partisanship and ideology. The table presents correlations between various demographic and media-use characteristics with the major correlates of COVID vaccination intention in both 2019 and 2021 as well with conspiracy mindset at both time points. It is evident that those with a conservative political bent in 2018 also endorsed conspiratorial thinking more strongly in 2021 than in 2019 (0.314 vs. 0.106). In addition, those who relied on conservative media were more likely to report conspiratorial thinking in 2021 (0.227 vs. 0.033 for traditional conservative media and 0.253 vs. 0.113 for ultra-conservative media). In addition, both Democrats and Republicans displayed changes in relation to conspiratorial thinking, with Democrats less attracted and Republicans more so. There were not dramatic changes in relation to use of other media, with users of both mainstream print and TV less likely to display conspiratorial thinking at both times. These changes suggest that the politization of the pandemic along with dissemination of conspiracy theories differentially affected the conspiratorial thinking of those on either side of the political spectrum.

The changes in correlations with the mindset were also accompanied by changes in other correlates of COVID vaccination. The most dramatic shifts occurred for vaccine misinformation, with Democrats reducing their endorsement of misinformation from 0.196 in 2019 to − 0.261 in 2021 and Republicans increasing their endorsement from − 0.100 in 2019 to 0.213 in 2021. Similar shifts were observed for conservatives and those using conservative media. Users of more mainstream media tended to reduce their belief in vaccine misinformation. Similar patterns were observed for perceptions of the risks of COVID compared with measles, with greater perceptions of risk for COVID associated with less misinformation. There was very little shift in trust of health authorities or willingness to take the vaccine for the flu.

The associations between mediating beliefs and various demographic characteristics also were observed. Respondents with greater education and income were more rejecting of misinformation in 2021 than in 2019, suggesting that the pandemic reversed some of the vaccination resistance associated with those differences in 2019. Similarly, older and female respondents became less accepting overall of misinformation in 2021. Black and Hispanic respondents reported more belief in misinformation in 2021 than 2019, while Evangelical respondents did not exhibit much change across time.

## Discussion

In this research, we interviewed a large national panel of US adults about vaccines in 2019 before the current COVID-19 pandemic and again early in 2021, shortly after the presidential election and the FDA emergency use authorization of vaccines against COVID-19 in the US.

Consistent with our hypotheses, we found that conspiratorial thinking in 2019 predicted distrust of health authorities and other indicators of vaccination hesitance in 2021 better than did 2019 trust and acceptance of misinformation alone. Tendencies toward conspiratorial thinking were related across time despite differences in measurement and were also highly related to factors associated with vaccination hesitancy in 2019 (Table 3), indicating that this form of thought was already associated with a wide range of beliefs opposed to vaccination even before the COVID pandemic. This pattern is consistent with other research that has found strong links between conspiratorial thinking and vaccination hesitancy in both the US and other countries^17,30,47^.

Our measure of conspiratorial thinking in 2021 adds further evidence that such thinking is driven by deep distrust of political and other institutions of authority. Not surprisingly, research has found that conspiratorial thinking is related to distrust of science in general^18,48,49^. As seen in Fig. 2, earlier conspiratorial thinking predicted a decline in trust which was greater than the carryover in trust from 2019 (− 0.54 vs. 0.27). In addition, conspiratorial thinking predicted belief in novel pandemic conspiracy theories (0.56) while earlier trust did not.

As noted by Pierre^30^, persons with a conspiracy mindset are likely to be attracted to information that supports their distrust. Accordingly, those with a conspiratorial mindset also avoided the use of mainstream news outlets that tended to support established health authorities and were more reliant on politically conservative and alternative health sources that questioned health authorities and advanced COVID-specific conspiracy theories. They were also much more likely to accept various forms of misinformation about vaccination, potentially supporting their anti-vaccination tendencies.

Unlike prior findings that distinguished COVID-specific from more general vaccination misinformation beliefs^16^, we found that both misinformation and conspiracy theories about COVID-19 vaccines were assimilated with all types of misinformation about vaccines. For example, the belief that COVID-19 vaccines “will change your DNA” loaded just as highly on the overall vaccination misinformation factor as other more general anti-vaccination beliefs. The conspiracy belief that the “pharmaceutical industry created the coronavirus to increase sales of its drugs and vaccines” also loaded on the misinformation factor. These findings are consistent with a review concluding that social media posts often link conspiracy theories about vaccines with various forms of misinformation about them^14^. These patterns also suggest that COVID specific beliefs were anchored in skepticism toward vaccines that predated the current pandemic.

Aside from conspiracies about vaccines, other conspiracy theories posited that the extent and severity of COVID-19 infections were exaggerated by some to harm President Trump’s reelection prospects. These beliefs were more directly associated with reduced perception of the threat of the disease, which also tended to be associated with less acceptance of COVID-19 vaccination. This pattern has been observed previously^21^ and is likely a result of downplaying the seriousness of the pandemic by the sitting president and conservative media^50,51^.

We also found evidence that the political polarization of the pandemic influenced persons with different political leanings to either greater acceptance or rejection of conspiratorial thinking. Although there was no evidence of overall increase in conspiratorial thinking over time, users of conservative media expressed more acceptance of conspiratorial thinking in 2021 than they had in 2019. Similar divergences were observed for Democrats and Republicans, with the former becoming less accepting of conspiratorial thinking while the latter becoming more so. Differences in acceptance of vaccination misinformation paralleled those relations. Although not directly tested by the structural model, it is clear that political ideology was positively related to conspiracy thinking in 2019 (r = 0.37) as well as to the use of both types of conservative media (0.33 and 0.21), with all three indirectly related to less overall support for COVID vaccination (− 0.23). These patterns suggest that conspiratorial thinking can be enhanced or reduced during periods of political conflict in which political leaders appeal to conspiratorial theories that are then disseminated by partisans or partisan-aligned media. Although it is possible that the differences between our time 1 and time 2 measures of conspiracy mindset were partly responsible for the differences in their relations with political identities, we found the same patterns in relation to vaccination misinformation which was closely related to conspiratorial thinking.

Our findings also speak to concerns that a conspiracy mindset is nothing more than a summary of beliefs in various conspiracies^44^. The mindset was not only predictive of distrust of health authorities but also predicted use of media that promulgated novel conspiracy theories about the pandemic. Indeed, use of conservative media in 2021 was more associated with the mindset than it was in 2019, suggesting that people who use politically-slanted media can become even more accepting of the mindset if it is supported by those media. Nevertheless, questions remain about the precursors of the mindset: Are certain individuals more prone to adopt it and what kinds of experiences predispose to its development?

Importantly, the level of accurate knowledge about vaccination increased for some in our panel between time 1 and time 2. As seen in Table 5, people with more education and income were more likely to endorse misinformation in 2019 that focused primarily on the MMR vaccine than when the items included vaccines for COVID in 2021, suggesting that the efforts to control the pandemic changed some previous skeptics into vaccine acceptors. A similar pattern was observed for age. These findings suggest that many of those who came to support COVID-19 vaccination were previously more resistant to vaccination, such as one population most vulnerable to it—the elderly (see Table 5).

### Limitations

Our panel in 2021 tended to be less nationally representative than it was when it was first created by Amerispeaks. While this over-represented older, more educated and wealthier respondents, the sample was comparable in terms of gender, religious identity, and political ideology, the latter two of which were strongly related to vaccination intentions. All analyses controlled for demographic and ideological differences, increasing the likelihood that our model of the influence of conspiratorial thinking was free from confounds with those characteristics. At time 2, our sample was comparable in terms of reported vaccination intentions for COVID-19 as assessed in other national surveys at the time^52^, adding further evidence that the findings are reflective of the factors that were at work in influencing vaccination intentions early in 2021.

We had different measures of conspiratorial thinking at the two waves, and this may have limited our ability to draw conclusions about differences in the overall levels of this mindset across time. However, we were able to identify differences in how the mindset correlated with other measures across time, including belief in misinformation and political ideology.

Although our findings were limited to the US, studies in Germany^53^ and Croatia^54^ also found that differences in support for COVID preventive actions were linked with contemporaneous conspiratorial thinking and were explained by differences in trust of the government. Thus, the phenomenon we isolated may not be limited to the US.

### Conclusions and policy implications

The major implication of our findings is that efforts to encourage acceptance of COVID-19 vaccines will hinge not only on providing information about their efficacy and safety from sources audience members find credible^55^ but also improving knowledge of vaccination in general^16^. Some strategies toward this goal include reducing uncertainty about misinformation since knowledge-based interventions may be more effective in targeting this outcome than in changing strong beliefs in misinformation^56^. Increasing transparency about the potential harms of vaccines has been found to increase trust in health authorities^57^. Discrediting or undercutting belief in conspiracy theories that undermine vaccination acceptance will also be important. Highlighting statements from presumed conspiracy promoters (e.g., Donald Trump supporting vaccination) has been found to increase vaccination rates^58^.

## Materials and methods

### Survey sample

APPC conducted six waves of surveys with a panel randomly selected from the NORC Amerispeaks panel^59^ of adults ages 18 + (N = 3000) starting in September 2018 and ending in October 2019. The Amerispeaks panel is a national probability sample that uses both online and telephone surveys to reach a representative cross-section of the US population. The surveys focused on knowledge and attitudes toward vaccines in general but with a focus on the measles and flu vaccines^8,60^. Respondents were queried about their assessment of misinformation about the safety and harms of vaccines, trust in health authorities, their uptake of the flu vaccine, and tendencies toward conspiratorial thinking. Because this process yielded a broad database of information about people’s vaccine hesitancy prior to the COVID pandemic, we were able to use it to test our hypotheses about the influence of those factors on acceptance of vaccination for COVID-19 following their emergency use authorization. The survey was deemed exempt from regulatory review by the University of Pennsylvania Institutional Review Board which conforms to the Declaration of Helsinki. In addition, all respondents gave informed consent to participate in the surveys at each wave of the study.

We invited the 1422 respondents who were still in the panel at the fifth wave of the study (April 2019) to complete a follow-up survey in late December 2020 and January 2021. The 4th and 5th waves (March and April 2019) provided responses for the analysis of vaccination beliefs and conspiratorial thinking. The first wave in 2018 provided information about religious identity, and the second, also in 2018, provided information about use of alternative health media and political party affiliation. A total of 1243 (87%) completed the follow-up (see Table 1 for demographic characteristics). The survey was also offered in Spanish, and 28 respondents (2.3%) completed it using that language. This sample tended to be older, more educated, racially white, and with higher income than the original panel at the first wave in 2018. However, the gender distribution as well as religious identity and political ideology were comparable. We used listwise deletion to study the follow-up sample rather than attempting to impute the missing cases, because imputation could overestimate carry-over from earlier waves. We also controlled for demographic differences in all analyses.

### Survey items

Tables 2, 3, 4, 5 have the wording of the items that were used to assess the major predictors of vaccination in 2019 and 2021.

#### Misinformation about the safety and efficacy of vaccination

In both years, we asked a variety of questions regarding the safety and unfounded fears of vaccines in general. In both surveys, we asked whether vaccines are a cause of autism in children and whether vaccines contain toxins such as antifreeze. We also sought an assessment of receipt of childhood vaccines on the recommended schedule and whether vaccines in the US were safe. Other items referred to the effectiveness of the MMR vaccine (2019) and whether getting a vaccination for the flu will give one a serious case of the disease. Some items referred specifically to misinformation about COVID vaccination, such as whether the COVID vaccine will give one COVID. While these were specific to the COVID pandemic, they factored along with the more general misinformation items.

#### Trust in health authorities

At both times, we asked about the trustworthiness of various health authorities, such as the CDC, the pharmaceutical industry, and primary care provider.

#### Covid vaccine conspiracy theories

At the 2021 survey, we asked about two conspiracy theories that were directly related to the vaccines that had recently been authorized on an emergency basis. One referred to whether the pharmaceutical industry secretly created the virus and the other to whether vaccines developed by Bill Gates might secretly be tracking their recipients.

#### Covid conspiracy beliefs

At the 2021 survey, we asked about acceptance of four conspiracy theories circulating in the US^61^ including that the virus was created by the Chinese government as a bioweapon.

#### Covid health threats

In the 2021 survey, we assessed various beliefs about the personal threat of the disease, such as whether the respondent was worried about contracting the virus or whether engaging in otherwise normal activities such eating in a restaurant would heighten risks to health. In the 2019 survey, we asked similar questions about the threat of measles.

#### Conspiratorial thinking

We measured conspiratorial thinking in two ways. In the 2019 survey, we asked about popular conspiracies, such as those that attributed the attacks on 9–11 to the US government or cast fluoridation of water as a plot to dump hazardous waste^17,40^. These items have been found to be highly related to measures of more generic conspiratorial thinking^41^, such as to whether our lives are controlled by plots hatched in secret places. In the 2021 survey, we assessed conspiratorial thinking in this way, using a scale that has been shown to predict vaccination intentions in the US^16,32^. Both measures are subject to response biases reflecting the undesirability of accepting conspiracies^46^. We identified these biases in the structural model with a method factor that allowed the items to be related apart from the underlying factor in the model.

#### Receipt of the flu vaccine

In both years, we asked whether the respondent either had or intended to receive the vaccine for the 2020/2021 season or had planned to get it for the upcoming flu season of 2019/2020. This served as a behavioral indicator of hesitancy to another recommended vaccine that has been found to predict hesitance toward vaccination for COVID-19^16^.

#### Media use

In the 2021 survey, we asked how much information respondents got from a variety of news and commentary sites, such as the mainstream television stations and social media^21^. We specifically asked about conservative media, such as Fox News, which had been linked with greater belief in COVID conspiracies during the first year of the pandemic^22^. In 2021, we also asked about newly emerging ultra-conservative media, such as Newsmax and The Gateway Pundit. Except for ultra-conservative media platforms, our 2018 and 2021 media measures were the same.

#### Use of alternative health media

Because conspiratorial thinking has been linked to use of alternative health aids^18–20^, we included information assessed in 2019 about whether respondents used any of three prominent types of alternative health media. Because use of the three tended to be highly inter-related, we took the first principal component, which accounted for 64% of the variance, as a summary score for this source of information.

#### Intention to receive the Covid vaccine

At the 2021 survey, we asked “When a no-cost vaccine that is approved by the Food and Drug Administration, also known as the FDA, to protect people from the coronavirus, also known as COVID-19, becomes available to you, how likely, if at all, would you be to get vaccinated? Responses were recorded from “Not at all likely”^1^ to “Very likely”^4^.

#### Political ideology

In both 2018 and 2021, we asked for placement on the liberal to conservative political spectrum: “Generally speaking, would you describe your political views as?” with responses going from Very liberal^1^ to Very conservative^5^ in order to control for differences in ideology that might be confounded with media use. In 2018, we also asked for political party identification.

#### Demographic characteristics

We included various personal characteristics that were related to vaccination acceptance, including gender, age, education, household income, and racial-ethnic identity (see Table 1). All characteristics were related to intentions to receive the COVID-19 vaccine by *X*^2^ tests, *p* < 0.001.

### Statistical analysis

We first conducted exploratory factor analyses to determine whether our classifications of items were consistent with the data. Those factors were then tested to confirm the measurement model using confirmatory factor analysis as described in the Results. Once a satisfactory measurement model was identified, we then used a structural equation model to test the major hypotheses that conspiratorial thinking in 2019 could predict COVID-19 vaccination intention in 2021 as mediated by trust in health authorities, beliefs in misinformation, concerns about the health effects of the infection, COVID conspiracy beliefs, and intention to receive the flu vaccine in 2021. Furthermore, we tested the alternative hypothesis that trust and misinformation as assessed in 2019 could predict subsequent mediators better than conspiratorial thinking in 2019.

We also tested the hypothesis that prior conspiratorial thinking would predict greater use of media that have been associated with growth in pandemic conspiracy beliefs and less use of media that have been associated with lower levels of those beliefs, relations we previously observed using the measure of generic conspiratorial thinking in 2021^16,32^. In this analysis, we also had a measure of use of alternative health media in 2019 that we included to determine whether this use of media could explain use of conservative media by conspiracy believers. Finally, we examined changes in correlations between demographic, media use, and political indicators between 2019 and 2021 to see if the politicization and heightened focus on vaccination changed how those differences were related to beliefs about vaccination and conspiratorial thinking.

The program Mplus was used to fit models^62^. Bootstrapping with 1000 samples was used to define 99% confidence intervals (CIs) for all model parameters, and standard goodness of fit indices were used to assess model fit^63^. Parameters that did not reach the 90% CI were dropped to enhance the parsimony of the final models. Although there was little missing data, Mplus uses full-information maximum likelihood methods to impute missing scores.

## Data Availability

The dataset analyzed in the current study is available from the corresponding author upon reasonable request.
